# Clinical outcomes of Ti-Ni shape-memory patella concentrator combined with cannulated compression screws in the treatment of C2 and C3 patella fracture: a retrospective study of 54 cases

**DOI:** 10.1186/s12891-020-03536-3

**Published:** 2020-07-31

**Authors:** Chen Yao, Jie Sun, Jiancheng Wu, Zhenyu Zhou, Fan Liu, Ran Tao, Yafeng Zhang

**Affiliations:** Department of Orthopaedics, The Affiliated Hospital of Nantong University, Nantong City, Jiangsu Province PR China

**Keywords:** Shape-memory patella concentrator, Comminuted patella fracture, Fixation technique, Clinical outcomes

## Abstract

**Background:**

Ti-Ni shape-memory patella concentrator (TNSMPC) has been designed as an alternative approach for fixation of patella fracture, which has some advantages like higher hardness, higher tenacity, better wearing resistance, excellent corrosion resistance and desired histocompatibility. The present study was to investigate the efficiency of TNSMPC combined with cannulated compression screws in the treatment of comminuted patella fractures.

**Methods:**

Between January 2014 and December 2017, 54 patients of C2 and C3 patella fractures underwent open reduction and internal fixation with TNSMPC combined with cannulated compression screws. All the patients got standard postoperative rehabilitation programs and were regularly followed up for at least 12 months after the operation. X-rays, knee functions and life quality were evaluated during the follow-up.

**Results:**

All the patients achieved bone healing and recovery of knee function with low incidence of complications according to outcomes of X-rays and questionnaires. The average operation time and blood loss during surgery were 77.5 ± 25.12 min and 24.25 ± 4.70 ml respectively. The Knee Outcome Survey Activities of Daily Living Scale, the range of motion and the 36-item short-form heath survey after the operation were all improved. According to the Bostman’s criteria, the excellent to good rate was 92.6%.

**Conclusion:**

TNSMPC combined with cannulated compression screws is an effective internal fixation method for C2 and C3 patella fracture with excellent clinical outcomes. In addition, the operation does not increase extra technique difficulty or tissue damage relatively, which is worth promotion.

## Background

The patella is the biggest sesamoid bone inside the human body, located between the quadriceps tendon and the patella tendon. As an important module in the knee extension system, the patella can function as a lever and increase the moment of quadriceps, enabling the extension of the knee and helping maintain the stability of knee joint [1]. The incidence of patella fracture in young people (18–49 years) is 0.01% [2]. What’s more, the incidence of patella fractures in adult including elder people is as high as 1.5%, accounting for 10% of all fractures [3]. Comminuted patella fractures (AO/OTA 34C2 and 34C3) are common challenges for orthopedic surgeons. Anatomical reduction and stable fixation are standard management to restore disrupted extension system and articular surface. Regardless of what kind of treatment was applied, there are still some patients who suffered poor outcomes from those comminuted patella fractures [4, 5].

While the operative techniques of patella fractures have undergone many changes over the past few decades, anterior tension band remains the most widely accepted method [6–8]. It can transfer tension into pressure force to achieve dynamic compression between fragments, allowing early joint motion. However, the tension band still has many disadvantages [9, 10]. The traditional tension band is composed of smooth Kirschner wires (K-wire) and stainless wires. The smooth K-wires cannot provide direct fragmentary compression and the fracture may separate when the knee extends [11]. In addition, when the tension band is working, the stainless wires and K-wires may have stress break, wearing, loosening, migration and skin irritation. Some orthopedic surgeons have presented different modified tension band methods to avoid the shortcomings of traditional tension band, such as cannulated screws combined with stainless wires or cannulated screws combined with titanium cables. However, the early failures of tension band were still reported in the literatures with the rate of 12 to 30%, especially in comminuted fractures [12, 13].

**Fig. 1:**
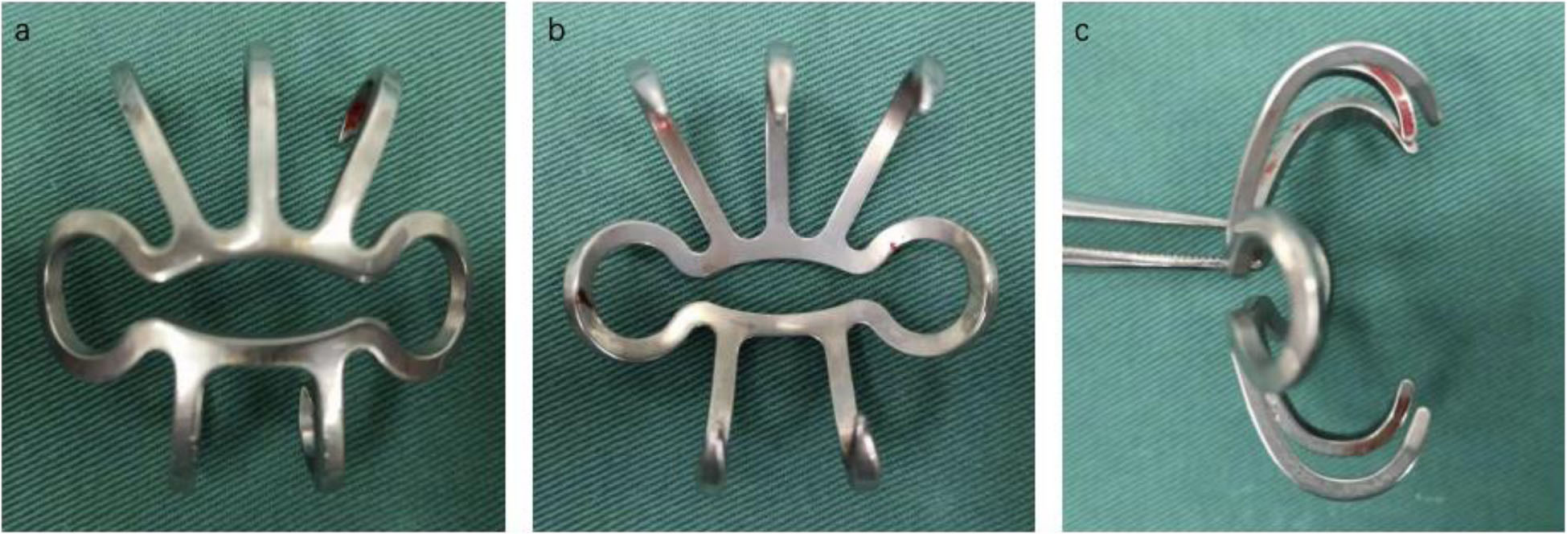
Diagram of the special-designed Ti-Ni shape-memory patella concentrator (SEEMINE, Lanzhou China, TN) for the fixation of patella fractures

Titanium-nickel (Ti-Ni) alloy is an one-way shape-memory alloy, which can be contoured in low temperature and reconverted to the previous shape in high temperature. Compared with traditional K-wires, the Ti-Ni shape-memory alloy has higher hardness, higher tenacity and better wearing resistance. What’s more, it has excellent corrosion resistance and desired histocompatibility [14, 15]. Based on these advantages, some Chinese factories have designed and manufactured patella concentrator out of Ti-Ni shape-memory alloy as an alternative approach for fixation of patella fractures. (Fig. 1).

During the past several years, we applied the special-designed Ti-Ni shape-memory patella concentrator (TNSMPC) combined with cannulated screws in the treatment of C2 and C3 patella fractures and conducted complete follow-up. In this paper, we presented our operation techniques. Additionally, we retrospectively collected and analyzed the existing clinical data of follow-up, evaluating clinical outcomes of this method.

## Materials and methods

### Patients

**Fig. 2:**
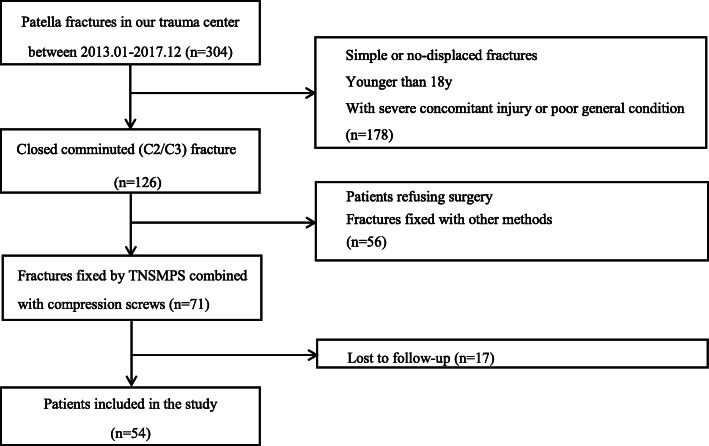
Flowchart of participants through the study

The retrospective study was conducted in Trauma Unit of the Department of Orthopaedics, the Affiliated Hospital of Nantong University, and approved by the Clinical Research Ethics Committee of the Institution. All works complied with the principles in the declarations of Helsinki. Between January 2014 and December 2017, all patients who came to our institution with patella fractures was evaluated for this study according to the special protocol shown in Fig. 2. The written informed consent was obtained from all patients. Inclusion criteria were: comminuted patella fracture (AO/OTA 34C2 and 34C3), indication for surgery (broken of knee extensor mechanism, fracture displacement of more than 3 mm, cartilage step-off of more than 2 mm), application of Ti-Ni shape-memory patella concentrator (TNSMPC) combined with cannulated screws, aged 18 years and above at the time of injury. Exclusion criteria were: simple or nondisplaced fracture with indication for conservative treatment (fracture displacement of no more than 3 mm or cartilage step-off of less than 2 mm), aged younger than 18 years, function limitation of the knee or other severe medical conditions before injury, multiple concomitant injuries of the ipsilateral leg or other systems, lost of follow-up.

### Perioperative management

After hospitalization, the injuried knee was temporarily fixed with splints in extension to release the pain. Standard anterior-posterior and lateral view X-rays were proceeded. Computerized tomography scan and three-dimensional reconstruction were performed for severely comminuted and displaced patella fractures. All fractures were classified according to AO/OTA classification system. Pre-operative function evaluation was gathered from medical documents. Open reduction and internal fixation were performed in 5 days since injury. Quadriceps contraction exercises and continuous passive motion (CPM) were carried out the day after operation. Partial weight bearing and active range-of-motion (ROM) exercises with a mobilizable brace were encouraged 1 week after operation. Full weight bearing walk was allowed 6 weeks after operation.

### Operation techniques

All patients got the operation under general anesthesia and supine position. The tourniquet was applied during the operation with 300 mmHg pressure. Anterior longitudinal incision was carried out to expose the fracture. The fractures were reduced and temporarily fixed with reposition forceps and guide pins of cannulated screws, and the reduction quality was checked by C-arm fluoroscopy simultaneously. If the reduction of the fracture was acceptable, cannulated screws with appropriate length were screwed in to fix the fracture fragments and provide direct fragmentary compression. The pre-patella bursa was sutured. TNSMPC was contoured in 0 °C sterilized normal saline and clasped the patella. The superior and inferior claws was respectively embedded into the tendon around the upper and lower patella pole, like a talon catching a prey. The concentrator was reconverted to the previous shape in 37 °C sterilized normal saline after implantation, which provided mechanical effects as a tension band. Stability of fracture fixation was confirmed with the knee in flexion of at least 90 degrees. Position of internal fixation and ROM of the joint were checked as well before closing the incision.

### Follow-up assessment

Follow-up assessment was performed at 3, 6, and 12 months after the operation, including X-rays and knee function. Patients’ life quality was evaluated at 12 months after the operation. The Knee Outcome Survey Activities of Daily Living Scale (KOS-ADL), ROM measurement, Bostman score and 36-item Short-form heath survey (SF-36) were applied in the process. The standards for clinical and bone healing of fracture healing were as the following: there was no local tenderness, no local tapping pain and patients can raised the injuried leg with the knee in extension easily; X-rays showed the fracture line is blurred and continuous callus passed through the fracture line.

### Statistical analysis

Statistical analysis was performed with the Graphpad Prism 7. Mogorov-Smirnov test was used to evaluate normal distribution. The normal distribution is expressed by mean (± standard deviation), while the non-normal distribution is expressed by median (25th and 75th percentiles) or number (percentage). The student t test and chi-square test were applied to compare the continuous variables and categorical variables respectively between different groups. *P* value of less than 0.05 was considered statistically significant.

## Results

Finally, 54 patients who accepted open reduction and internal fixation with TNSMPC combined with cannulated screws for comminuted patella fracture were evaluated in this study. All the cases were close fractures, without other concomitant injuries. Baseline demographic and clinical characteristics of the patients were summarized in Table 1. There were 31 males and 23 females. The average age of patients were 54.11 ± 11.24 years (range: 28–83 years). The average body mass index (BMI) was 23.02 ± 4.30 kg/m^2^. Thirty-three fractures happened in the left side and 21 fractures happened in the right side. The injury mechanisms included traffic accidents (28 patients), sports (10 patients), works (9 patients) and activities in daily life (7 patients). According to the AO/OTA classification system, there were 23 C2 fractures and 31 C3 fractures. The average hospitalization was 14.67 ± 4.40 days. The average operation time was 77.5 ± 25.12 min and the average blood loss during the operation was 24.25 ± 4.70 ml (range: 20-40 ml).

**Table 1:** Baseline demographic and clinical characteristic of patients

Characteristic	Description
Age, years (mean ± SD)	54.11 ± 11.24
Gender, n%	
Male	31 (57.4)
Female	23 (42.6)
Side of injury	
Left	33 (61.1)
Right	21 (38.9)
BMI, kg/m^2^ (mean ± SD)	23.02 ± 4.30
Mechanism of injury, n%	
Daily activity	
Work	9 (16.7)
Traffic accident	28 (51.9)
Sports injury	10 (18.5)
Activities in daily life	7 (13.0)
OTA classification	
C2	23 (42.6)
C3	31 (57.4)
Hospital stay, day (mean ± SD)	14.67 ± 4.40
Operation time, min (mean ± SD)	77.50 ± 25.12
Blood loss, ml (mean ± SD)	24.25 ± 4.70

**Fig. 3:**
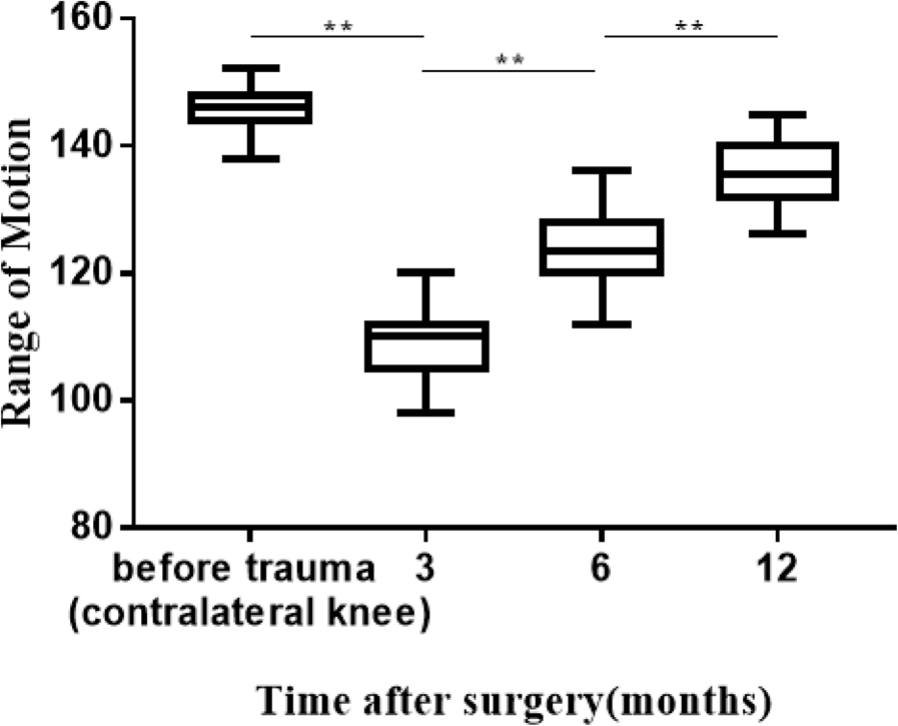
Histogram of the ROM for extension-flexion at follow up. The ROM for extension-flexion of the injured knee improved significantly from 108.91° ± 4.95° after 3 months to 124.28° ± 5.09° after 6 months and 136.11° ± 4.42° after 12 months, close to the uninjured contralateral knee (146.42° ± 3.43°) **: t-test, *P* < 0.0001.

**Fig. 4:**
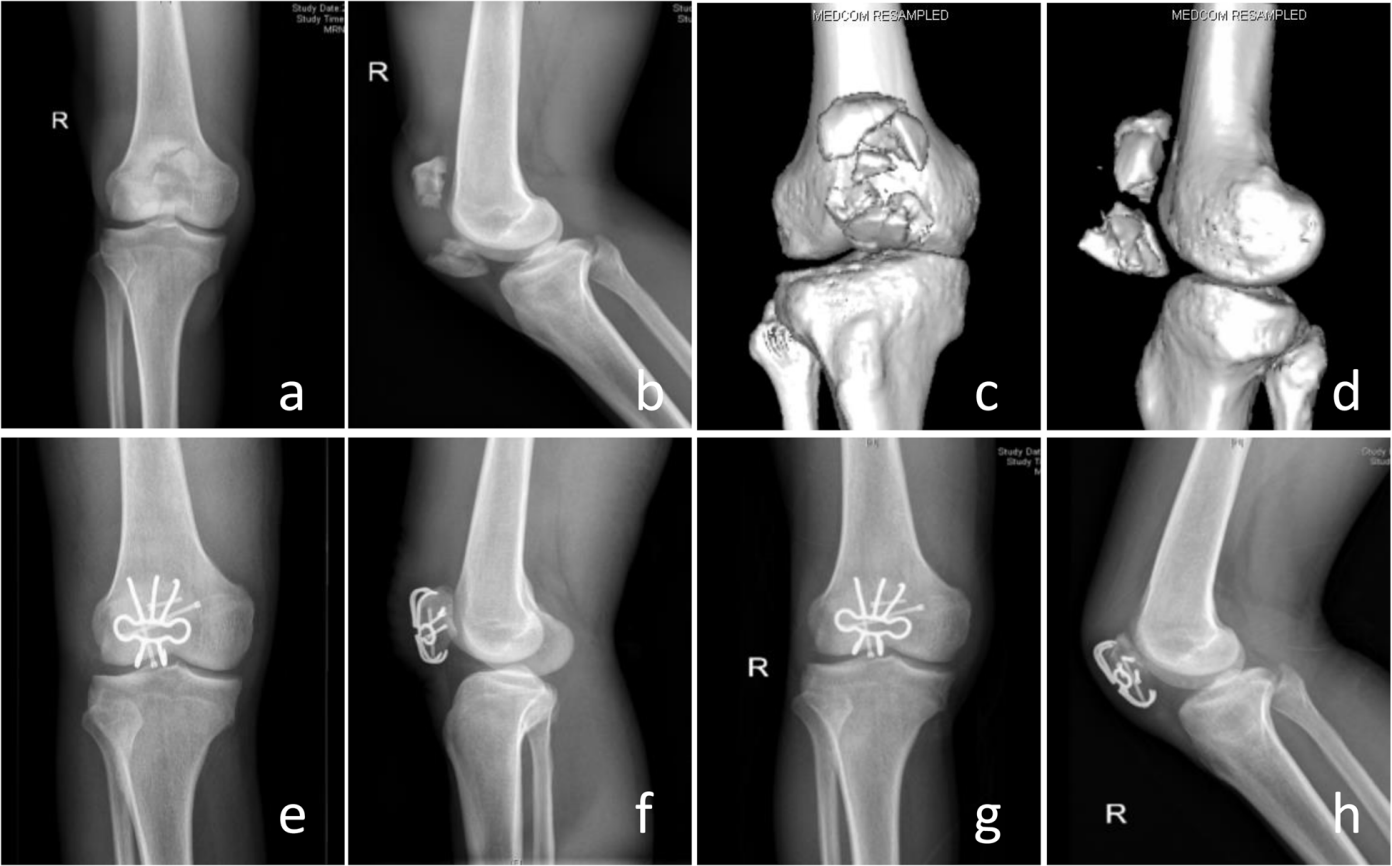
Comminuted fracture (AO/OTA 34C3) of the patella in a 48-year-old male patient after a car accident (**a-d**). Open reduction and internal fixation with TNSMPC and compression screws were performed. X-rays immediately after surgery (**e, f**) and 3 months later (**g, h**) showed desirable outcomes

All the patients got clinical healing 3 months after operation and bone healing 6 months after operation according to the results of radiographic and clinical evaluation. No broken, loosening, migration and skin irritation of the internal fixation were detected, which means excellent performance and histocompatibility of TNSMPC. The ROM for extension-flexion of the injuried knee improved significantly (*P* < 0.0001) from 108.91° ± 4.95° 3 months after operation to 124.28° ± 5.09° 6 months after operation and 136.11° ± 4.42° 12 months after operation, and closed to the uninjuried contralateral knee (146.42° ± 3.43°) (Fig. 3). The KOS-ADL was evaluated at least 6 months after the operation. The results showed only a few patients had symptoms severely influencing their daily activities (Table 2). Kneeling and squatting were limited for several patients and other functions were not difficult for patients (Table 3). SF-36 survey 1 years after the operation indicated the quality life of the patients were almost restored (Table 4). The results of role physical evaluation were relatively lower than other items, which indicated patients may need appropriate physical rehabilitation training respectively after the operation. According to the Bostman score, among all the 54 patients, 38 patients were excellent, 12 cases were good. The Fig. 4 showed a demo case.

**Table 2:** KOS-ADL evaluation (Symptoms) 1 year after the surgery

	I do not have the symptom in my knee	I have the symptom, but it does not affect my activity	The symptom affects my activity slightly	The symptom affects my activity moderately	The symptom affects my activity severely	The symptom prevents me from all daily activity	NO answer
Pain	22	9	14	4	5	0	0
Stiffness	30	4	8	3	5	0	4
Swelling	28	8	6	4	4	0	4
Slipping	20	9	8	4	9	0	4
Buckling	19	9	11	5	8	0	2
Weakness	29	6	6	6	2	2	3

**Table 3:** KOS-ADL evaluation (Functional limitations) 1 year after the surgery

	Activity is not difficult	Activity is minimally difficult	Activity is somewhat difficult	Activity is fairly difficult	Activity is very difficult	I am unable to do the activity	No answer
Walk	25	13	8	3	3	0	2
Go up stairs	17	18	5	9	3	0	2
Go down stairs	12	18	8	11	3	0	2
Stand	27	12	5	2	5	0	3
Kneel on front	4	12	9	6	9	12	2
Squat	14	12	2	12	2	6	6
Sit with knee bent	22	11	12	5	0	0	4
Rise from a chair	20	12	6	8	3	0	5

**Table 4:** SF-36 health survey 1 year after the surgery

	All patients (*n* = 54)
Items	
PCS ^a^	63.84 ± 18.45
MCS ^a^	74.81 ± 14.29
PF ^b^	80 (65,80)
RP ^b^	25 (0,100)
BP ^b^	100 (74,100)
GH ^a^	65.19 ± 17.05
VT ^a^	68.06 ± 15.91
SF ^a^	76.39 ± 18.76
RE ^b^	100 (58.33,100)
MH ^a^	77.63 ± 6.97

*Abbreviations*: *PCS* physical component summary, *MCS* mental component summary, *PF* physical function, *RP* role-physical, *BP* bodily pain, *GH* general health, *VT* vitality, *SF* social function, *RE* role emotional, *MH* metal health

^a^: Values are presented as the mean ± SD, ^b^: Values are presented as the median (IQR)

## Discussion

The purpose of this retrospective case series was to evaluate the clinical outcomes of TNSMPC combined with cannulated compression screws in the treatment of comminuted patella fractures. The results revealed good clinical outcomes and few complications of this technique, which achieved high level of patient’s satisfaction. Due to the reliable fixation provided by the implants, patients were allowed to full weight bearing and rehabilitation after the surgery as soon as possible. All patients got bone healing 6 months after operation, with improved knee function, better life quality and few severe complications. In addition, the average operation time was 77.5 min and average blood loss was 25.5 ml, which means this operation method may not increase extra technique difficulties or tissue damages and it worth being promoted widely.

KOS-ADL and ROM are the most commonly used to evaluate the function of the knee after the surgery like arthroscopy, arthroplasty or fracture. The Bostman score reflects the patients’ subjective satisfaction to the treatments. Particularly, as a concise health questionnaire, SF-36 comprehensively summarizes the quality of life of the respondent from eight aspects: physiological function, physiological function, physical pain, general health, vitality, social function, role emotional and mental health. The vast majority of patients recovered significantly in terms of body function and quality of life after surgery in our study, while there are a small number of subjects who cannot kneel, squat or go down stairs easily after the surgery. We think it is a failure of patients to perform proper functional exercise after surgery and related to the fact that some patients themselves are too old and have poor mobility. Generally, the ROM of injured knee, function scores and life quality evaluation of patients indicated excellent recovery 1 years after surgery compared with the perioperative period.

**Fig. 5:**
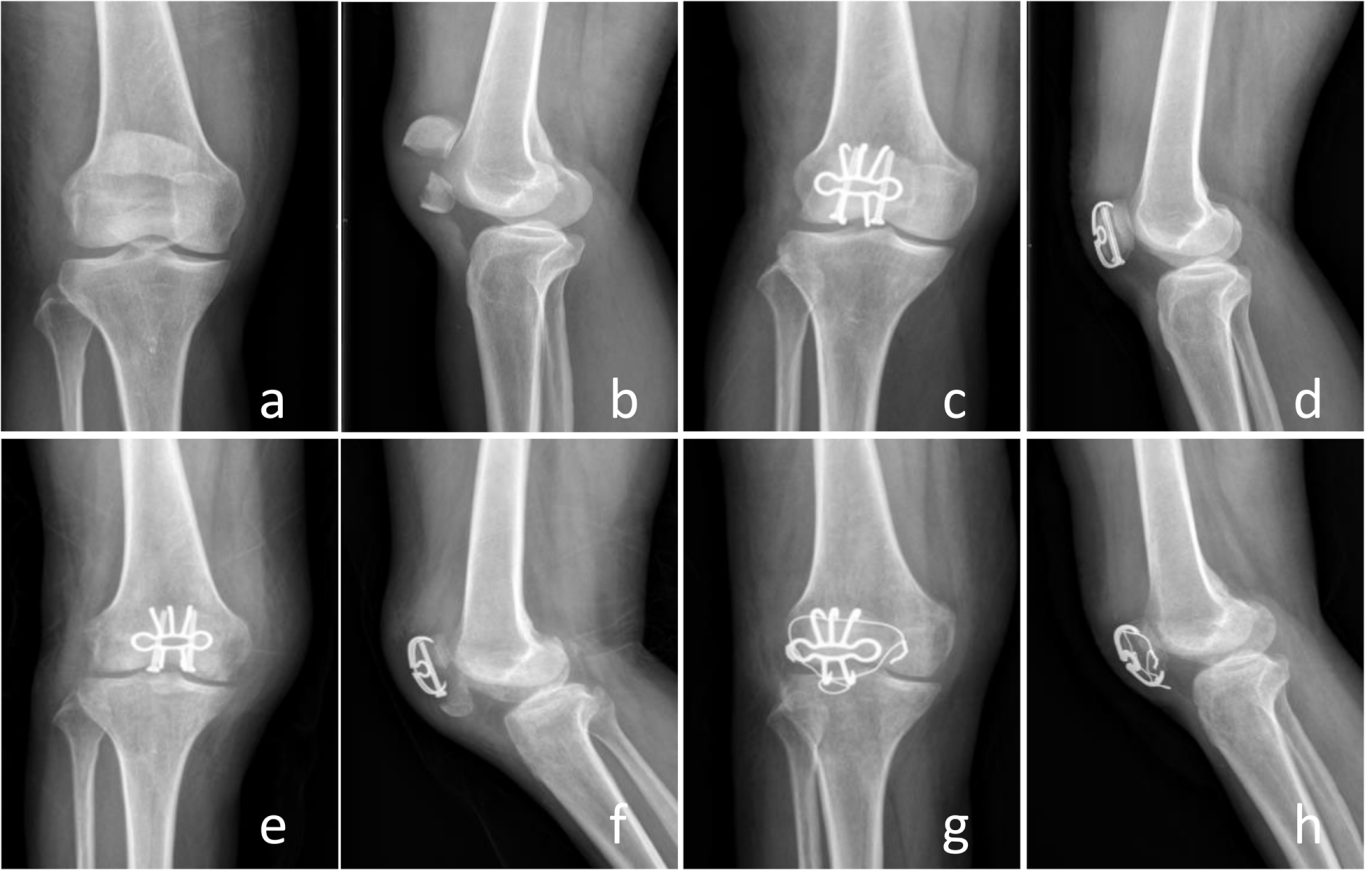
Transverse fracture of the patella (**a, b**) in a 55-year-old female patient treated with TNSMPC and compression screws (**c, d**). The internal fixation failed 6 weeks after the surgery (**e, f**). The screws were removed and the claw and additional circle wire fixation were applied for revision surgery **(g, h)**

Longitudinal K-wires combined with stainless wire in a figure-8 pattern was a classical fixation method for patella fractures and has been widely applied for decades. The cannulated compression screws can be applied to replace the K-wire without threads to provide direct fragmentary compression with the knee in extension [16]. However, the early failure of the internal fixation due to stress break, wearing, loosening, migration, electrolytic and skin irritation still exists, particularly for comminuted patellar fractures [12, 13]. To avoid intrinsic shortcomings of tension wiring, some new internal fixations have been designed and applied in clinical trials such as a fixed angle plate, a locking patella plate and TNSMPC in our study [17–19]. The TNSMPC applied in our study was designed according to the anatomical characteristics of patella and was made of Ti-Ni alloy with high excellent corrosion resistance and desired histocompatibility. The TNSMPC can provide similar or even stronger dynamic compression between fragments like anterior tension band and can avoid the intrinsic defects of anterior tension band. The shape of the concentrator fits for the patella very well and the material is friendly to the soft tissue, reducing risk of skin problems. In addition, Ti-Ni alloy has excellent corrosion resistance and mechanical properties, which can provide stable fixation for comminuted fractures and avoid early stress break, wearing, loosening or migration. In terms of operation techniques, the install of the concentrator is much easier than that of stainless wire and the learning curve is short. After contoured in 0 °C sterilized normal saline and installed on the patella, concentrator was reconverted in 37 °C normal saline and clasped the patella like a talon strongly, without any extra damage of soft tissue. Other new fixations, such as fixed-angle plating and locking plate, need extra screws to fix the plate on the patella and have limitations in osteoporotic patients or severely comminuted fractures [20]. However, the TNSMPC can fix the fracture independently by its claws and causes no extra bone loss, which can achieve excellent outcomes in osteoporotic or comminuted cases. The study conducted by Yutong Zhang et al., described another similar patella concentrator and reported desired outcomes in pole fracture or comminuted patella fractures [21]. Fracture displacement due to the loosening of the claw is a potential complication of the operation while it did not happen in cases with comminuted fractures. Figure 5 shows the failure of the internal fixation in a case with a simple transverse fracture and the revision surgery was performed by removing the screws, reducing the fracture and fixing it with the claw and additional circle wire. Factors related to the complications are not so clear, which may include choosing inappropriate size of the claw, malposition of the implants, poor bone quality, knee flexion too early after the operation and so on.

Several limitations of our study exist and deserve mention. Actually, this is a retrospective case series without a control group and we didn’t compare the outcomes of our method with other osteosynthesis techniques directly, which affected the evidence level. We only chose comminuted fractures according to rigid standard in a single trauma center, so the number of cases are limited. Finally, the time of follow up was not so long. In the future, more cases from multiple trauma center and results of long-term follow up will be collected.

## Conclusion

In summary, results of our study showed excellent outcomes of TNSMPC combined with cannulated compression screws in the treatment of C2 and C3 patella fracture. Without extra technique difficult or tissue damage, the TNSMPC and screws provide dynamic and static fragmentary compression respectively, ensuring stable fixation of comminuted patella fractures. Patients are allowed to early weight-bearing and rehabilitation without extra complications. As an alternative solution of traditional tension band, TNSMPC combined with cannulated compression screws is appropriate for comminuted patella fractures and worth spreading.

## Data Availability

The datasets used and/or analysed during the current study are available from the corresponding author on reasonable request.

## Abbreviations

BMI: Body mass index
CPM: Continuous passive motion
KOS-ADL: Knee Outcome Survey Activities of Daily Living Scale
K-wire: Kirschner wire
ROM: Range of motion
Ti-Ni: Titanium-nickel
TNSMPC: Ti-Ni shape-memory patella concentrator
PCS: Physical component summary
MCS: Mental component summary
PF: Physical function
RP: Role-physical
BP: Bodily pain
GH: General health
VT: Vitality
SF: Social function
RE: Role emotional
MH: Metal health
IQR: Interquartile range
